# Contralateral Selectivity of Upper-Limb Motor Pools via Targeted Stimulation of the Cervical Spinal Cord

**DOI:** 10.3390/biomedicines11020332

**Published:** 2023-01-24

**Authors:** Neil Fleming, Clare Taylor, Mark Etzelmueller, Conor Gill, Clodagh O’Keeffe, Nicholas Mahony, Richard B. Reilly

**Affiliations:** 1 Discipline of Anatomy, School of Medicine, Trinity College Dublin, D02 R590 Dublin, Ireland; 2 School of Engineering, Trinity College, The University of Dublin, D08 XW7X Dublin, Ireland; 3 Discipline of Gerontology School of Medicine, Trinity College, The University of Dublin, D02 R590 Dublin, Ireland; 4 Trinity Centre for Biomedical Engineering, Trinity College Dublin, D02 R590 Dublin, Ireland

**Keywords:** transcutaneous spinal cord stimulation, tSCS, cervical spine, upper limb, spinal cord injury

## Abstract

Transcutaneous spinal cord stimulation (tSCS) at the cervical level may facilitate improved upper-limb function in those with incomplete tetraplegia. While clinical trials are ongoing, there is still much debate regarding the transmission pathway as well as appropriate stimulation parameters. This study aimed to explore the extent to which cervical tSCS can induce mono-synaptic reflexes in discrete upper-limb motor pools and examine the effects of altering stimulus location and intensity. Methods: Fourteen participants with intact nervous systems completed two laboratory visits, during which posterior root-muscle reflexes (PRMRs) were evoked via a 3 × 3 cathode matrix applied over the cervical spine. An incremental recruitment curve at the C7 vertebral level was initially performed to attain resting motor threshold (RMT) in each muscle. Paired pulses (1 ms square monophasic with inter-pulse interval of 50 ms) were subsequently delivered at a frequency of 0.25 Hz at two intensities (RMT and RMT + 20%) across all nine cathode positions. Evoked responses to the 1st (PRMR_1_) and 2nd (PRMR_2_) stimuli were recorded in four upper-limb muscles. Results: A significant effect of the spinal level was observed in all muscles for PRMR_1_, with greater responses being recorded caudally. Contralateral stimulation significantly increased PRMR_1_ in *Biceps Brachii* (*p* < 0.05, F = 4.9, η2 = 0.29), *Flexor Carpi Radialis* (*p* < 0.05, F = 4.9, η2 = 0.28) and *Abductor Pollicis Brevis* (*p* < 0.01, F = 8.9, η2 = 0.89). Post-activation depression (PAD) was also significantly increased with contralateral stimulation in *Biceps Brachii* (*p =* 0.001, F = 9.3, η2 = 0.44), *Triceps Brachii* (*p* < 0.05, F = 5.4, η2 = 0.31) and *Flexor Carpi Radialis* (*p* < 0.001, F = 17.4, η2 = 0.59). Conclusions: A level of unilateral motor pool selectivity may be attained by altering stimulus intensity and location during cervical tSCS. Optimising these parameters may improve the efficacy of this neuromodulation method in clinical cohorts.

## 1. Introduction

Over the last decade, electrical stimulation of the spinal cord either via implanted (eSCS) or cutaneous (tSCS) electrodes has emerged as a viable neuromodulation approach for restoring a level of motor function in previously paralysed individuals [1,2,3,4]. Neuromodulation is broadly defined as any method aimed at inducing plasticity within the central nervous system (CNS) either via stimulation of afferent sensory pathways or via the use of efferent signals (such as EMG) to provide feedback [5]. Therefore, in order to consider tSCS a neuromodulation method, it must operate via stimulation of afferent sensory pathways and not direct stimulation of the efferent motor pools.

While the majority of clinical studies have focused on lumbothoracic tSCS for improving gait and standing, more recent studies have used cervical tSCS to improve grip strength, pinch strength and overall upper-limb function in individuals with chronic cervical spinal cord injury (SCI) [4,6,7,8]. Computational and experimental data from lumbo-thoracic stimulation suggest that tSCS activates medium-to-large-diameter sensory afferents within the dorsal roots [9,10,11], resulting in a monosynaptic reflex in the corresponding motor pools. Such responses are most commonly termed posterior root-muscle reflexes (PRMRs) [11]. By modulating reflex excitability at a spinal level, it is thought that tSCS may supplement tonic sensory and supraspinal inputs, leading to plasticity of existing locomotor and/or postural control circuitry in those with paralysis [12].

The trans-synaptic reflex transmission of tSCS is typically verified using paired stimuli delivered with a short (30–50 ms) inter-pulse interval, such that the 2nd response is attenuated or absent. When fired in such quick succession, large-diameter 1a afferents are unable to release sufficient neurotransmitters, resulting in post-activation depression (PAD) of the 2nd response [13]. By comparing the conditioning (PRMR_1_) and test (PRMR_2_) motor responses, PAD can, therefore, be quantified, allowing researchers to evaluate the extent to which tSCS engages afferent sensory pathways. While PAD has been widely confirmed in lumbo-thoracic tSCS [9,10,11], there is still debate regarding the reflex nature of tSCS applied at the cervical level. While de Freitas, Sasaki [14] argue a predominant reflex origin of tSCS delivered across multiple segments (C6-T1), Wu, Levine [13] demonstrated that preferential excitation of upper-limb 1a afferents is highly dependent on stimulus intensity and that both ventral and dorsal roots may be simultaneously excited.

Experimental data have also shown that discrete lower-limb motor pools can be selectively activated via rostro-caudal [15] and medio-lateral [16] changes in electrode position. More recently, de Freitas, Sasaki [14] confirmed bilateral selectivity of upper-limb motor pools via stimulation at the C6, C7 and T1 vertebral levels, with higher stimulus currents required to elicit responses in the distal motor pools responsible for hand and wrist function. Preferential activation of hand muscles was found from stimulation at the T1 level, but selectivity was not observed in more proximal muscles. However, the extent to which cervical tSCS can unilaterally select discrete motor pools remains to be elucidated. Considering the importance of unilateral motor control for precise upper-limb functional tasks such as reaching and grasping, it is important for researchers and clinicians to understand the interactions between stimulation parameters such as intensity and location in order to better administer this therapeutic method.

The primary aim of this study was, therefore, to evaluate the level of selectivity within upper-limb motor pools that can be attained via cervical tSCS. A secondary aim was to assess the transmission pathway for cervical tSCS, and a final aim was to evaluate the test–retest reliability of two commonly used stimulation characteristics [13,14]. We hypothesized that (similar to lumbo-thoracic tSCS [16]) ipsilateral stimulation would preferentially activate upper-limb motor pools and more caudal stimulation would preferentially activate more distal muscles controlling wrist and hand functions.

## 2. Materials and Methods

### 2.1. Study Design

**Figure 1:**
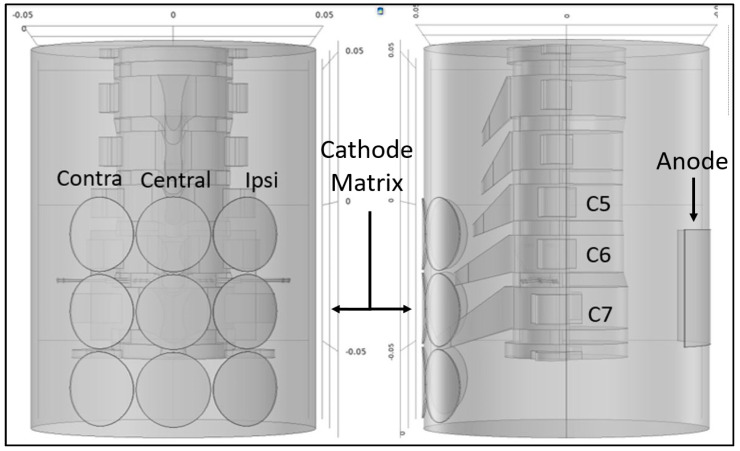
Graphical model of electrode array in the coronal (left) and sagittal (right) planes. A 3 × 3 cathode matrix was placed dorsally at the approximate levels of C5/6, C7 and C8 spinal nerves in contralateral, central and ipsilateral arrangements. A rectangular 5 × 10 cm anode was placed ventrally, with the caudal border of the electrode in line with the C7 spinous process.

A total of 14 participants (9 males and 5 females; aged 27 ± 4 years; height, 176 ± 86 cm; weight, 73.9 ± 12.5 kg) with intact nervous systems completed two test visits, during which tSCS was applied at various locations and intensities on the cervical spine. During Visit 1, a recruitment curve was conducted with a single stimulation configuration. During Visit 2, the same recruitment curve was initially conducted prior to the application of a 3 × 3 cathode matrix (Figure 1). A series of three paired pulses were subsequently applied at each location and at two intensities in a randomized order. The Faculty of Health Sciences Research Ethics Committee at Trinity College Dublin approved the experimental protocols (FREC: 2020110), which were conducted in accordance with the Declaration of Helsinki.

### 2.2. Protocol

Cathode electrodes (ø 3.2 cm^2^; Axelgaard, Fallbrook, CA) were placed in a 3 × 3 array centrally over the C5-C6, C6-C7 and C7-T1 intervertebral spaces and ~3.2 cm laterally on either side (see Figure 1). These locations were assumed to be the most proximal to the C5/6, C7 and C8 spinal nerves, respectively. A fixed anode (5 × 10 cm; Axelgaard, Fallbrook, CA, USA) was placed centrally on the anterior neck with the caudal border at the level of the C7 spinous process. During Visit 1 and Visit 2, intensity–response curves were first generated via incremental stimulation of the C7 central cathode. This test involved a series of three paired pulses (1 ms monophasic square wave, 50 ms IPI) delivered at 0.25 Hz with 5 mA increments from 10 mA up to 80 mA (or maximal tolerance). A maximal stimulation intensity of 80 mA is within the range typically employed in cervical tSCS studies [17]. Resting motor threshold (RMT) was subsequently determined as the lowest stimulus intensity at which PRMRs were consistently evoked in two or more muscles with 3 consecutive stimuli. The criterium for PRMRs was set as a peak-to-peak amplitude of 50 µV [13]. Following determination of RMT, individualised stimulation intensities for testing across the 3 × 3 cathode array were calculated (Lo = RMT, Hi = RMT + 20%). A series of three paired pulses (1 ms monophasic, IPI = 50 ms) were applied at a frequency of 0.25 Hz at each of the two stimulus intensities across all nine cathodal locations, in a randomised order, using a constant current stimulator (DS8R; Digitimer Ltd., Welwyn Garden City, UK). Heart rate and blood pressure data along with participant comfort levels were recorded throughout the experiment. A numeric rating scale (NRS) was used to evaluate participant comfort from 0 to 10 with 0 corresponding to “no pain” and 10 corresponding to “worst pain imaginable” [18]. Cardiovascular response was measured before and during stimulation protocols with an automated sphygmomanometer (M3; Omron Corporation, Kyoto, Japan).

### 2.3. Electromyography (EMG)

**Figure 2:**
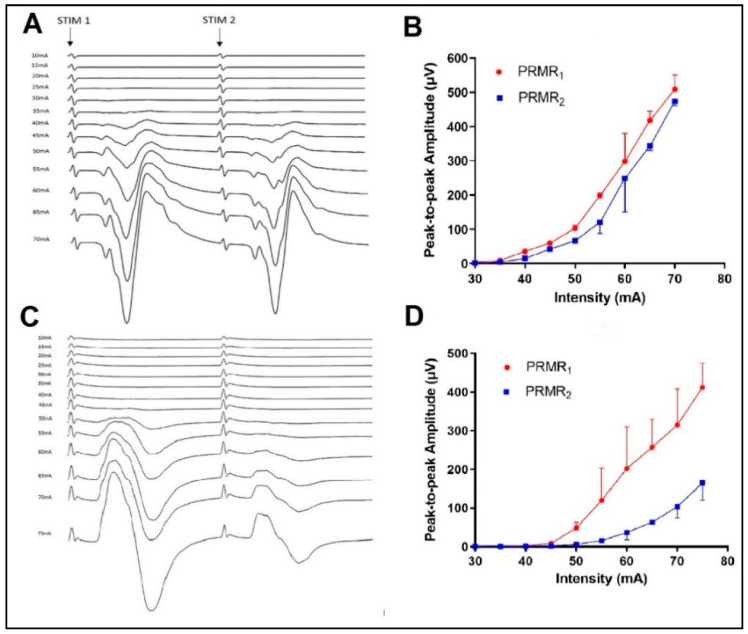
Incremental EMG traces (**A**,**C**) and corresponding intensity–response curves (**B**,**D**) of FCR muscle in two separate participants. Note the large variation in PRMR_2_ response between individuals ((**B**) vs. (**D**)).

Surface EMG recordings were recorded in *Biceps Brachii* (BB), *Triceps Brachii* (TB), *Flexor Carpi Radialis* (FCR) and *Abductor Pollicis Brevis* (APB) on the right arm using pairs of pre-gelled Ag/AgCl electrodes (Kendall, Mansfield, MA, USA). The skin sites were shaved and cleaned with isopropyl alcohol wipes prior to electrode application. Recording electrodes were placed centrally over the muscle belly with longitudinal alignment, in accordance with the SENIAM recommendations [19]. All recordings were conducted with the participants seated comfortably in a chair, arms supported symmetrically on both sides via arm rests and neck in a neutral position. EMG signals were acquired using Octal Bioamp integrated into a Powerlab 16/35 system (ADInstruments, Sydney, Australia). Signals were recorded at 10 kHz, amplified (CMMR > 60 dB), bandpass-filtered (10–500 Hz) and digitized. Evoked responses were recorded into LabChart (V8; ADInstruments, Oxford, UK) and subsequently exported to Matlab (MATLAB 2020b: The MathWorks Inc., Natick, MA, USA) for processing and analysis. Data were initially processed to remove any DC offset, and bandpass-filtered between 20 and 500 Hz prior to stimulus artefact removal using a customised curve-fitting programme. The peak-to-peak response amplitude was then quantified in all EMG traces in the range of 6–40 ms after both 1st (PRMR_1_) and 2nd (PRMR_2_) pulses (see Figure 2) and averaged over three consecutive paired pulses for all nine cathode locations and two stimulation intensities.

### 2.4. Data Processing

Outcome variables quantified for the purposes of comparing stimulus intensity and location were PRMR_1_ and PAD. PRMR_1_ was normalised to the maximal PRMR recorded in any location or intensity throughout Visit 2. PAD was measured in each muscle as previously described [13].

PAD (%) = [1 − PRMR_2_/PRMR_1_] × 100 (1)

For the purposes of evaluating the reliability of stimulus intensity characteristics, we compared two of the most recently cited definitions [13,14]. Resting motor threshold (RMT) was defined as the minimal current (in mA) that elicited a PRMR > 50 uV [13], and PAD_max_ (i.e., the maximal suppression of PRMR_2_) was quantified as the maximal PAD attained within a muscle from the associated C7 intensity–response curves [14]. For the purposes of evaluating the test–retest reliability of cervical tSCS stimulation characteristics, RMT, PAD_max_ and the current at which PAD_max_ occurred (X_D_) were quantified in each muscle. These variables were subsequently compared across visits.

### 2.5. Statistical Analysis

Data for each muscle were assessed for normality with Kolmogorov–Smirnov tests. Non-normally distributed data were transformed into Gaussian distributions with log or antilog transforms prior to further analysis. The effects of spinal level (C5/6, C7 and C8 spinal nerves), lateral location (contralateral, central and ipsilateral) and stimulation intensity (RMT and RMT + 20%) on the magnitude of PRMR_1_ and PAD were evaluated using 3 × 3 × 2 repeated measures ANOVA (row × column × intensity). Violations of sphericity were corrected with Greenhouse–Geisser. A *p*-value of *p* < 0.05 inferred statistical significance at all times, and Eta squared (η2) was used for quantifying overall effect sizes. Subsequent post hoc testing of lateral location (contralateral, central and ipsilateral) and stimulation intensity (RMT and RMT + 20%) at each spinal level was conducted with 3 × 2 repeated measures ANOVA (column × intensity). Pairwise comparisons were conducted using Bonferroni’s correction, with Cohen’s D (d) quantifying the effect size. The following descriptors were applied to all effect sizes: trivial, 0–0.19; small, 0.2–0.49; moderate, 0.5–0.79; large, >0.8).

The test–retest reliability of RMT, PAD_max_ and X_D_ was evaluated and compared across muscles with intraclass correlation and Bland–Altman analysis. The relative reliability of the measures was expressed as intraclass correlation coefficients (ICCs), and absolute reliability was expressed in terms of technical error of the measure (TEM), minimal detectable change (MDC_95_) and 95% limits of agreement (LOAs). Munro’s descriptors for reliability coefficients were used to describe the degree of reliability: 0.00 to 0.25—little if any; 0.26 to 0.49—low; 0.50 to 0.69—moderate; 0.70 to 0.89—high; and 0.90–1.00—very high [20].

## 3. Results

### 3.1. Test–Retest Reliability of Stimulus Characteristics

A comparison of the test–retest reliability of two commonly used stimulation characteristics is presented in Table 1. Both stimulation characteristics have previously been used to evaluate cervical tSCS [13,14]. RMT, in general, appears more the more reliable stimulation characteristic, with higher ICC and lower TEM and MDC_95_ than PADmax for each muscle evaluated.

### 3.2. Effects of Spinal Level on PRMR_1_

A moderate effect of the spinal level was observed for PRMR_1_ response in BB, TB and FCR, while a large effect was observed in APB (see Table 2 for *p*-values and effect sizes). In all muscles, PRMR_1_ was significantly greater at the most caudal level of C8 than that at either the C5/6 or C7 level.

Comparing spinal levels, PRMR_1_ response in BB was significantly greater at C8 than those at C5 (*p* < 0.0001, t = 7.24, d = 0.47) and C7 (*p* < 0.01, t = 4.74, d = 0.21), while response at C7 was significantly greater than that at C5/6 (*p* < 0.001, t = 5.43, d = 0.29). PRMR_1_ response in TB was significantly greater at C8 than those at C5/6 (*p* < 0.0001, t = 8.26, d = 1.03) and C7 (*p* < 0.01, t = 4.16, d = 0.55), while response at C7 was significantly greater than that at C5/6 (*p* < 0.001, t = 5.99, d = 0.64). PRMR_1_ response in FCR was significantly greater at C8 than those at C5/6 (*p* < 0.0001, t = 5.46, d = 0.75) and C7 (*p* < 0.001, t = 5.46, d = 0.75), while response at C7 was significantly greater than that at C5/6 (*p* < 0.01, t = 5.08, d = 0.80). PRMR_1_ response in APB was significantly greater at C8 than those at C5/6 (*p* < 0.0001, t = 10.75, d = 1.25) and C7 (*p* < 0.0001, t = 11.22, d = 0.99), while response at C7 was significantly greater than that at the C5/6 level (*p* < 0.001, t = 5.21, d = 0.76).

### 3.3. Effects of Stimulus Intensity and Lateral Location on PRMR_1_

**Figure 3:**
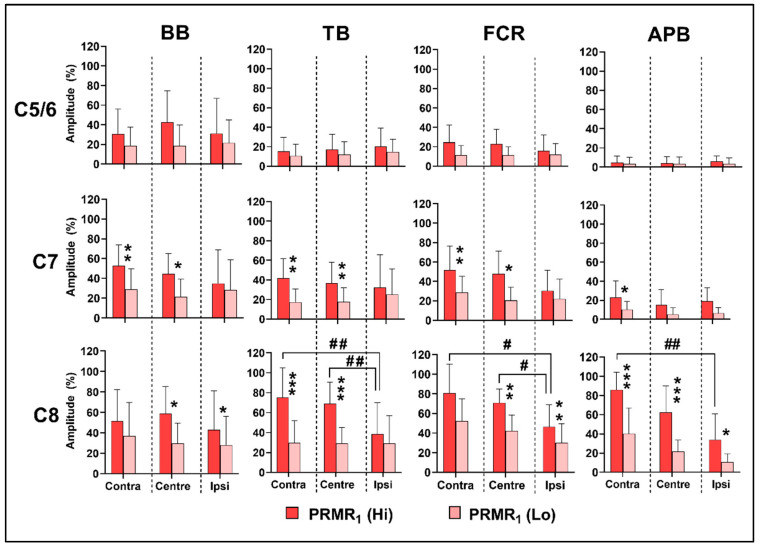
Group mean ± SD amplitude of PRMR_1_ across spinal levels (C5/6, C7 and C8), lateral locations (contralateral, central and ipsilateral) and intensities (Hi = dark; Lo = light) in BB, TB, FCR and APB. Asterisks infer significantly greater PRMR_1_ response comparing high with low stimulus intensity (* *p* < 0.05; ** *p* < 0.01; *** *p* < 0.001). Hash symbols infer significantly lower PRMR_1_ response at ipsilateral stimulus location (# *p* < 0.05; ## *p* < 0.01).

Stimulus intensity had a large overall effect on PRMR_1_ in BB, FCR and APB, while a moderate overall effect was observed in TB (see Table 2 for *p*-values and effect sizes). Regarding the spinal levels, a moderate intensity effect was observed in BB at the C5/6 (*p* < 0.001, F = 20.29, η2 = 0.63), C7 (*p* < 0.0001, F = 35.86, η2 = 0.75) and C8 levels (*p* < 0.01, F = 16.19, η2 = 0.57). Similarly, in TB, moderate intensity effects were observed at the C5/6 (*p* < 0.01, F = 14.57, η2 = 0.55), C7 (*p* < 0.0001, F = 36.65, n2 = 0.75) and C8 levels (*p* < 0.0001, F = 44.00, η2 = 0.79). In FCR, moderate intensity effects were observed at the C5/6 (*p* < 0.001, F = 19.65, η2 = 0.62), C7 (*p* < 0.0001, F = 44.71, η2 = 0.79) and C8 levels (*p* < 0.001, F = 23.3, η2 = 0.66). A small intensity effect was observed in APB at C5/6 (*p* < 0.05, F = 8.46, η2 = 0.41), while a moderate effect (*p* < 0.0001, F = 30.05, η2 = 0.71) and a large effect (*p* < 0.0001, F = 117.63, n2 = 0.91) were observed at the C7 and C8 levels, respectively. In all muscles and at all spinal levels, PRMR_1_ amplitude was greater using the highest intensity stimulation (see Figure 3).

The lateral location of the cathode electrode had a small effect on APB, BB and FCR, respectively (see Table 2 for *p*-values and effect sizes). A small interaction effect between intensity and lateral location was also observed at the C7 level in BB (*p* < 0.05, F = 5.21, η2 = 0.3) and at the C7 (*p* < 0.01, F = 6.1, η2 = 0.05) and C8 levels in TB (*p* < 0.01, F = 6.3, η2 = 0.04). These interactions were evident as a progressive increase in PRMR_1_ amplitude as the cathode electrode was moved from the ipsilateral to the contralateral position, only within high-intensity stimulations (see Figure 3). Among the distal muscles, a small effect of cathode location was observed in FCR (*p* < 0.01, F = 23.3, η2 = 0.43), and a moderate effect observed in APB (*p* < 0.001, F = 14.8, η2 = 0.55), at the C8 level only. In both muscles, the contralateral location of the cathode electrode resulted in significantly greater PRMR_1_ amplitude; however, the differences were only observed when using the highest stimulation intensity (see Figure 3).

### 3.4. Effects of Spinal Level on Reflex Transmission

No significant effects of the spinal level were observed for PAD in BB, TB or FCR. In contrast, ABP showed a small effect of the spinal level (see Table 2). Post hoc testing highlighted that PAD at the C8 level was significantly greater than that at the C5/6 level only (*p* < 0.01, t = 4.56, d = 0.98). No other pairwise differences were observed.

### 3.5. Effects of Lateral Location and Intensity on Reflex Transmission

**Figure 4:**
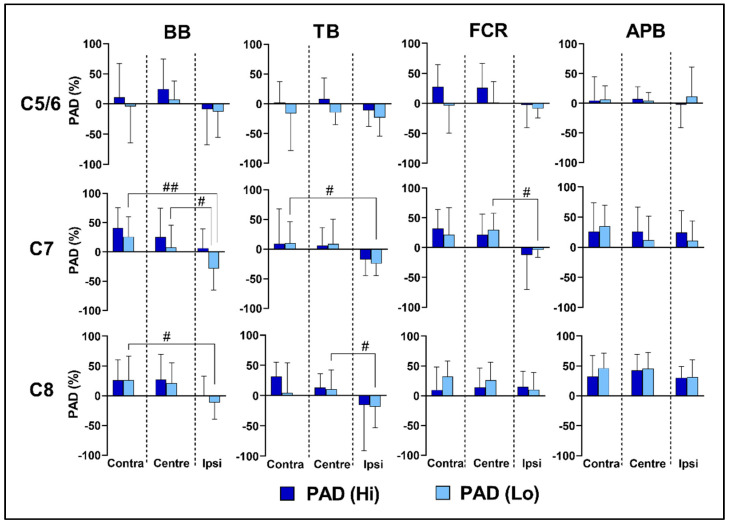
Group mean ± SD PAD across spinal levels (C5, C7 and C8), locations (contralateral, central and ipsilateral) and intensities (Hi = dark blue; Lo = light blue) in BB, TB, FCR and ABP. Note that negative data infer potentiation rather than depression of PRMR_2_. Hash symbols infer significantly lower PAD response at ipsilateral stimulus location (# *p* < 0.05; ## *p* < 0.01).

The lateral location of the cathode electrode had a small overall effect on PAD responses in BB and TB and a moderate effect in FCR (see Table 2 for *p*-values and effect sizes). In BB, this lateral effect was small at the C7 (*p* < 0.001, F = 10.77, η2 = 0.47) and C8 levels (*p* < 0.001, F = 11.9, η2 = 0.49). Similarly, in TB, a small lateral effect was observed at the C7 (*p* < 0.05, F = 5.24, η2 = 0.30) and C8 levels (*p* < 0.05, F = 8.03, η2 = 0.40). A small lateral effect was also observed in FCR at the C7 level (*p* < 0.05, F = 7.38, η2 = 0.38). In all three muscles, contralateral stimulation increased the level of trans-synaptic reflex transmission (see Figure 4). However, the lateral location of the cathode had no effects on PAD in APB at none of the spinal levels.

An effect of stimulation intensity was observed in the largest proximal muscles, with both BB (*p* < 0.05) and TB (*p* < 0.001) demonstrating greater reflex responses at higher stimulus intensity. However, this effect was only evident at the C7 level in BB (*p* < 0.05, F = 6.89, η2 = 0.36) and at the C5/6 level in TB (*p* < 0.05, F = 5.26, η2 = 0.30), and no significant pairwise effects of stimulation intensity were observed at none of the lateral locations in neither BB nor TB (see Figure 4).

### 3.6. Tolerability and Cardiovascular Response

Blood pressure and heart rate were assessed throughout testing for fluctuations. Systolic blood pressure across the cohort did not differ comparing measures taken before and during stimulation (124 ± 78 vs. 124 ± 79 mmHg, respectively). Similarly, no significant changes in diastolic blood pressure were observed (78 ± 10 vs. 79 ± 10 mmHg). Resting heart rate recorded before (69 ± 13 beats·min^−1^) and during (67 ± 12 beats·min^−1^) stimulation did not significantly differ. One participant’s heart rate increased by more than 20% during the experiment, but it was not sustained and returned to normal levels upon the subsequent assessment. No symptoms were displayed. All other participants remained within normal levels throughout the duration of the experiment. Discomfort ranged from NRS 2 to 8 during stimulation (median NRS = 5). In all cases where NRS was greater than 5, discomfort did not last once stimulation was ceased. In some cases, redness was observed over the electrode site following cessation of stimulation; however, this was asymptomatic and resolved within 30 min. One participant experienced low back pain on the day following Visit 1 and was withdrawn from the study; however, this adverse event may have been due to an unrelated muscle strain during a gym session that took place on the same day.

## 4. Discussion

A growing body of literature has reported therapeutic effects of cervical tSCS for upper-limb function in those with paralysis [4,5,6,7]. These studies have all applied a fixed stimulation frequency, intensity and electrode configuration. However, experiments using eSCS suggest that spatiotemporal selectivity of motor pools [1] may be necessary to maintain appropriate phase-dependant modulation of proprioceptive inputs to the spinal cord [21]. Considering that tSCS requires current to be passed through a variety of tissues with differing electrical impedance values (skin, subcutaneous fat, muscle and bone), the level of selectivity possible and the precise neural structures activated remain unclear, particularly for cervical tSCS. In the current study, we unexpectedly demonstrated that contralateral cathodal stimulation of the cervical region at the level of the C8 spinal nerve not only preferentially activated discrete upper-limb motor pools but also enhanced reflex transmission pathways. These findings may help to provide more targeted delivery of tSCS for future therapies aimed at retraining unilateral motor function for tasks such as reaching and grasping.

### 4.1. Selectivity of Upper-Limb Motor Pools

The main finding of this study was that cervical tSCS administered with a contralateral cathode configuration preferentially activated upper-limb motor pools. A significant medio-lateral effect was observed for PRMR_1_ in TB, FCR and APB at the C8 level (Figure 3), with stimulation on the contralateral side increasing the response in these motor pools. Contralateral stimulation also enhanced trans-synaptic reflex transmission to BB, TB and FCR motor pools, as highlighted by the significantly increased PAD recorded in these muscles (Figure 4). Previously, Calvert, Manson [16] reported preferential activation of lower-limb motor pools using lumbothoracic tSCS. However, they observed greater evoked responses using an ipsilateral cathode configuration, the opposite of what we observed. More recently, Oh, Steele [22] reported ipsilateral selectivity of upper-limb motor pools using cervical tSCS, again the opposite of what we observed.

Our contrasting findings with respect to Oh, Steele [22] may be explained by differences in stimulating electrode positions, particularly the location of the anode. While our anode was located at a site proximal to the cathode matrix (see Figure 1), their anodes were placed bilaterally above the anterior iliac crest. Comparing our findings to Calvert, Manson’s [16], it is likely that regional variations in the vertebral architecture, along with differing curvature and orientation of the spinal nerves emanating from the intervertebral foramina, may explain the regional differences in motor pool selectivity between cervical and lumbothoracic tSCS. Among the many tissues and structures that current must pass through, bone offers the greatest electrical impedance [23]. It, therefore, stands to reason that the vertebral architecture is likely to influence the passage of current and thus the discrete neural tissues stimulated. Cervical vertebrae possess longer laminae, curved in a convex rather than concave direction. They also lack prominent transverse and (with the exception of C7) prominent spinous processes. In addition, the spinal nerves of the cervical region exit their respective intervertebral foramina at much greater angles than those of the lumbosacral enlargement and with fewer neural anastomoses [24]. The net effect of these anatomical differences is that cervical vertebrae may offer greater exposure of the intervertebral foramina and less shielding of the spinal nerves, particularly on the contralateral side.

In terms of the spinal level, research has already highlighted that upper-limb PRMR response increases significantly in APB when cathode location is moved caudally [14]. Our results support these findings and highlight that placement of cathodes at levels above C7 may be sub-optimal, even for muscles proximal to the elbow (i.e., BB and TB). This was unexpected, since BB and TB are innervated by musculocutaneous (C5-C7) and radial (C6-C8) nerves, respectively. Even in these proximal muscles, more caudal stimulation increased the evoked responses (see Figure 3). Stimulation at more caudal levels on the upper thoracic spine (T1-T2) may further enhance motor pool selectivity, particularly for the distal muscles controlling wrist and hand functions; however, the exploration of the upper thoracic region was beyond the scope of the current study. Future studies may further optimize motor pool selectivity by exploring more caudal cathode locations overlying the T1 spinal nerve.

### 4.2. Reflex Transmission of tSCS

A critical assumption regarding the use of tSCS as a neuromodulation approach is the preferential stimulation of large-diameter sensory afferents within the dorsal roots and reflex transmission to their corresponding motor pools in the ventral horn [14]. This has been demonstrated with paired stimuli (IPI 20–50 ms) applied in the lumbothoracic region [9,10,11]. The magnitude of PAD observed in the current study was highly variant among individuals and muscle groups (Figure 2) and, in general, lower than that previously reported for cervical tSCS [13,14]. Wu, Levine [13] reported PAD in APB of 50–60% when stimulated at the same stimulus intensities as in our study. Comparing the same motor pool and stimulation intensities, the highest level of PAD we observed in ABP was 46 ± 25% (contralateral stimulation over C8). The lower levels we observed may have been due to the IPI used, which was 10 ms longer in the current study. However, Hofstoetter, Freundl [25] reported 100% PAD in Soleus up to IPIs of 100 ms in a neurologically intact cohort. We, therefore, chose a 50 ms IPI since our entire cohort were neurologically intact (in contrast to Wu, Levine [13]). de Freitas, Sasaki [14] reported significant levels of PAD in upper-limb muscles across three spinal levels, with approximately 62%, 43% and 51% PAD being observed at the C6 spinal level, in BB, TB and FCR, respectively (visual interpolation of their Figure 4). They used the same 50 ms IPI as that in the current study; however, the stimulation intensity was higher. Our results provide further support that both dorsal and ventral rootlets are likely stimulated during cervical tSCS. They also highlight the complex interaction between stimulus location and intensity required in order to maximise trans-synaptic reflex response in upper-limb motor pools.

In some cases, potentiation rather than depression of PRMR_2_ was observed (Figure 4). This was particularly evident when stimulation was delivered ipsilaterally and may represent evidence of the activation of other neighbouring neural structures, for example, pre-synaptic activation of cortico-motoneural projections, which are known to greatly facilitate spinal excitability, particularly in hand and wrist muscles [26]. Primates possess a well-developed cortico-spinal system with projections from the motor cortex directly synapsing with upper-limb motor pools [27]. Indeed, the ratio of motor to 1a afferent inputs appears to be related to the functional demands of discrete motor pools [27], with greater motor cortical control over intrinsic wrist and hand muscles. This difference based on functional demand is most clearly observed in the lower limbs, with contrasting effects in the Tibialis Anterior and Soleus motor pools when TMS is applied alone [28] or in combination with tSCS [29]. Recently, Wecht, Savage [30] demonstrated that subthreshold tSCS paired with synchronous TMS facilitates convergent transmission to upper-limb motor pools. Perhaps the longer latency (50 ms) potentiation observed in the current study—when the cathode was placed ipsilaterally—may, therefore, represent evidence of pre-synaptic cortico-motoneural excitation.

Another possible explanation for the ipsilateral potentiation observed is the preferential activation of sensory pathways on the opposite side. It has been demonstrated that peripheral stimulation of the median nerve increases excitability of the motoneuronal pool on the contralateral side with long-lasting effects [31]. Since contralateral electrode placement in the current study appeared to stimulate more of those target neural structures (i.e., large-diameter sensory afferents), perhaps those same structures on the opposite side enhance spinal excitability when the stimulating electrodes are placed ipsilaterally.

Regardless of the neural pathways involved, it is worth acknowledging that the magnitude of motor cortical and afferent inputs differs across discrete motor pools and limbs, and these differences may be functionally specific. If spinal excitability is functionally dependent on a specific interplay of cortical and sensory afferent inputs, then practitioners should consider what neural structures can and should be stimulated with cervical tSCS. Inducing plasticity within this spinal circuitry with the electrical stimulation of sensory reflex pathways may not serve much purpose if the underlying motor pools are listening more attentively for other inputs, such as cortico-spinal pathways. Perhaps, the general observation of low-level PAD with cervical tSCS simply reflects a lower level of the 1a afferent input to these upper-limb motor pools when compared with the extensive 1a afferent input observed in lower-limb antigravity muscles such as Soleus [26].

### 4.3. Effects of Intensity on PRMR_1_ and PAD

As expected, higher stimulation intensities significantly increased PRMR_1_ response in all muscles (see Figure 3 and Table 2). However, the effect of stimulation intensity was less consistent regarding reflex transmission. While higher intensity stimulation significantly increased PAD in both BB (*p* < 0.05) and TB (*p* < 0.001), the distal muscles favoured lower-intensity stimulation (see Figure 4). This is in agreement with previous research [13,14], which also reported that increasing stimulus intensity is likely to progressively activate motor pools directly via ventral rootlets. Our results further highlight the importance of “fine-tuning” stimulus intensity, in order to optimise the activation of sensory reflex pathways.

### 4.4. Test–Retest Reliability of Stimulation Indices

Both RMT [13] and XD [14] have recently been used as a means for characterizing stimulation via cervical tSCS. Both measures demonstrated moderate-to-high repeatability in the current study; however, RMT, in general, appeared the most reliable stimulation characteristic (see Table 1). The absolute reliability data may be useful for future interpretations of electrophysiological assessment and therapeutic prescription. For example, de Freitas, Sasaki [14] compared RMT and XD across three spinal levels. Their analysis revealed significant differences in both parameters. However, the current study reports MDC_95_ ranging from 11 to 17 mA for RMT and from 14 to 25 mA for XD, depending on the motor pool examined. Caution should, therefore, be exercised when interpreting differences or changes in these variables. If the difference in mean data is less than the minimal detectable difference, such results may be statistically significant but not clinically meaningful.

### 4.5. Study Limitations

The results of this study should be interpreted in the context of several study limitations. Firstly, the data were collected from a neurologically intact cohort. It is possible that the level of selectivity and reflex excitability may differ between neurologically intact individuals and those with SCI. Hofstoetter, Freundl [25] previously reported a significant overall effect of the neurological status on the recovery cycles of PRMR responses in the Soleus motor pool. However, the differences observed between cohorts were observed over longer IPIs, of 150–1000 ms, with little effects being observed in 40, 60 or 80 ms IPI. More recently, Wu, Levine [13] observed no differences in the magnitude of PAD in APB between those with SCI and a neurologically intact cohort. Nonetheless, the neurological status of the current cohort limits any large-scale clinical extrapolation of the findings. Secondly, this study focussed on examining PRMR and PAD response via tSCS alone. The authors acknowledge that an understanding of the precise neural structures stimulated using this approach remains to be fully elucidated. Future studies that combine this stimulation approach with paired stimuli delivered via TMS and/or peripheral nerve stimulation could allow a more detailed examination of the role of motor-cortical and/or sensory pathways to the responses observed to be performed.

## 5. Conclusions

This study reports both medio-lateral and rostro-caudal selectivity of upper-limb motor pools via tSCS. Unexpectedly, we observed enhanced stimulation of upper-limb motor pools with the contralateral positioning of the cathode electrode. Similar to other studies of cervical tSCS, the level of reflex transmission achieved in upper-limb motor pools was highly variable among and within individuals, and it was lower than those of previous reports on lumbothoracic tSCS, highlighting the importance of optimising stimulation parameters such as stimulus location and intensity. We hope these findings will assist future clinical studies that combine targeted tSCS with upper-limb rehabilitation exercise in order to ensure that appropriate neuromodulation takes place.

## Figures and Tables

**Table 1:** Group means (SDs) of stimulation characteristics derived from Visit 1 and Visit 2 intensity/response curves. Relative (ICC) and absolute (TEM and 95% LOAs) measures of reliability are also presented for resting motor threshold (RMT), maximal post-activation depression (PAD_max_) and the current at which PAD_max_ occurred (X_D_).

RMT (mA)	Visit 1	Visit 2	ICC	TEM	MDC_95_	±95% LOAs
BB	42 (6)	42 (10)	0.72	4	11	−12/+12
TB	40 (8)	41 (9)	0.70	5	14	−16/16
FCR	45 (10)	45 (9)	0.63	6	17	−14/13
APB	39 (8)	41 (10)	0.77	4	11	−14/10
**PAD_max_ (%)**						
BB	80 (10)	73 (21)	0.24	11	30	−32/46
TB	60 (14)	50 (30)	0.10	22	61	−52/71
FCR	61 (16)	57 (25)	0.23	18	50	−47/56
APB	50 (23)	61 (17)	0.48	15	42	−48/26
**X_D_ (mA)**						
BB	54 (11)	51 (8)	0.61	6	17	−13/19
TB	48 (10)	49 (11)	0.78	5	14	−14/13
FCR	50 (12)	48 (10)	0.59	7	19	−17/22
APB	49 (14)	47 (17)	0.61	9	25	−25/29

**Table 2:** Results of three-way ANOVA for comparison of stimulus intensity, spinal level and lateral cathode location in PRMR_1_ and PAD. In all cases where significant lateral effects are reported, greater responses were observed with contralateral cathode arrangement. In all cases of spinal levels, greater responses were observed at the most caudal level (level of C8 spinal nerve).

	PRMR_1_				PAD			
	BB	TB	FCR	APB	BB	TB	FCR	APB
**Lateral Effect**	F = 4.9 *p* = 0.012 η2 = 0.29	-	F = 4.9 *p* = 0.035 η2 = 0.28	F = 8.9 *p* = 0.001 η2 = 0.43	F = 9.3 *p* = 0.001 η2 = 0.44	F = 5.4 *p* = 0.012 η2 = 0.31	F = 17.4 *p* = 0.000 η2 = 0.59	-
**Spinal Level**	F = 39.7 *p* = 0.000 η2 = 0.77	F = 43.4 *p* = 0.000 η2 = 0.79	F = 39.8 *p* = 0.000 η2 = 0.77	F = 107.0; *p* = 0.000 η2 = 0.90	-	-	-	F = 7.7 *p* = 0.003 η2 = 0.39
**Intensity**	F = 65.6 *p* = 0.000 η2 = 0.85	F = 44.9 *p* = 0.000 η2 = 0.79	F = 65.6 *p* = 0.000 η2 = 0.85	F = 145.6 *p* = 0.000 η2 = 0.92	F = 6.5 *p* = 0.026 η2 = 0.35	F = 22.6 *p* = 0.000 η2 = 0.65	-	-

## Data Availability

All data included in this study can be obtained upon request via email to the corresponding author.
